# Deconfounded and debiased estimation for high-dimensional linear regression under hidden confounding with application to omics data

**DOI:** 10.1093/bioinformatics/btaf400

**Published:** 2025-07-14

**Authors:** Zhaoyang Li, Yahang Liu, Kecheng Wei, Yongfu Yu, Guoyou Qin, Zhongyi Zhu

**Affiliations:** Department of Biostatistics, School of Public Health, Fuda n University, Shanghai, 200032, China; Department of Biostatistics, School of Public Health, Fuda n University, Shanghai, 200032, China; Department of Biostatistics, School of Public Health, Fuda n University, Shanghai, 200032, China; Department of Biostatistics, Shanghai Stomatological Hospital & School of Public Health, Fudan University, Shanghai, 200032, China; Department of Biostatistics, School of Public Health, Fuda n University, Shanghai, 200032, China; Department of Statistics and Data Science, Fudan Univers ity, Shanghai, 200094, China

## Abstract

**Motivation:**

A critical challenge in observational studies arises from the presence of hidden confounders in high-dimensional data. This leads to biases in causal effect estimation due to both hidden confounding and high-dimensional estimation. Some classical deconfounding methods are inadequate for high-dimensional scenarios and typically require prior information on hidden confounders. We propose a two-step deconfounded and debiased estimation for high-dimensional linear regression with hidden confounding.

**Results:**

First, we reduce hidden confounding via spectral transformation. Second, we correct bias from the weighted $\ell_{1}$ penalty, commonly used in high-dimensional estimation, by inverting the Karush–Kuhn–Tucker conditions and solving convex optimization programs. This deconfounding technique by spectral transformation requires no prior knowledge of hidden confounders. This novel debiasing approach improves over recent work by not assuming a sparse precision matrix, making it more suitable for cases with intrinsic covariate correlations. Simulations show that the proposed method corrects both biases and provides more precise coefficient estimates than existing approaches. We also apply the proposed method to a deoxyribonucleic acid methylation dataset from the Alzheimer’s disease (AD) neuroimaging initiative database to investigate the association between cerebrospinal fluid tau protein levels and AD severity.

**Availability and implementation:**

The code for the proposed method is available on GitHub (https://github.com/Li-Zhaoy/Dec-Deb.git) and archived on Zenodo (DOI: https://10.5281/zenodo.15478745).

## 1 Introduction

In observational studies, the lack of randomization often leads to the presence of hidden confounders, which are unobserved and associated with both the outcome and exposures. In medical research, conventional adjustment approaches, such as regression adjustment, matching, and propensity score methods, only control for known confounders and are unable to address hidden confounders (Vina and Lloret 2010, Hayes *et al.* 2020). Thus, these hidden confounders can result in biased estimates of coefficients between exposures of interest and the outcome. Meanwhile, with the expansion of data sources, such as omics, the issue of controlling hidden confounding in high-dimensional settings has garnered significant attention from researchers.

Some of the classic methods for deconfounding include instrumental variable regression (Gold *et al.* 2020), two-stage calibration method (Yang and Ding 2020), and latent confounder adjustment model (Wang *et al.* 2017). These approaches not only rely on prior knowledge of hidden confounders but are also usually inadequate for high-dimensional scenarios. Unlike these methods, spectral deconfounding estimation directly shrinks the hidden confounding through spectral transformation without requiring any prior information about hidden confounders. Ćevid *et al.* (2020) proposed to run the lasso method on the spectral transformed data where some leading singular values of the design matrix are shrunk by the spectral transformation matrix. Although mitigating the hidden confounding, the deconfounded estimator inherits the bias of the lasso method.

The lasso method is one of the most popular high-dimensional regression methods, where the $\ell_{1}$ penalty introduces a non-negligible bias (Tibshirani 1996, Fu and Knight 2000). To address this bias, various procedures have been developed (Fan and Li 2001, Zou 2006, Zhang 2010, Javanmard and Montanari 2014, Van de Geer *et al.* 2014, Zhang and Zhang 2014, Cai and Guo 2017, Javanmard and Lee 2020, Cai *et al.* 2023a, 2023b, Xia *et al.* 2023). Among these, debiased lasso approaches first obtain the lasso estimator and then correct its bias through low-dimensional projections (Zhang and Zhang 2014) or by inverting Karush–Kuhn–Tucker conditions (Javanmard and Montanari, 2014, Van de Geer *et al.* 2014). Nonetheless, the aforementioned approaches are limited to conventional linear regression models and fall short of addressing the hidden confounding.

To fill this gap, Guo *et al.* (2022) further debiased the deconfounded estimator by low-dimensional projections (Zhang and Zhang, 2014). Similarly, Sun *et al.* (2024) adopted an analogous deconfounding and debiasing procedure, differing primarily in using a different spectral transformation matrix. However, during debiasing, they both employed node-wise regression to construct projection vectors, which requires a sparse precision matrix assumption. This assumption is difficult to fulfill in practical studies due to the correlation between covariates. For example, in our real data application based on the Alzheimer’s disease neuroimaging initiative (ADNI) database, we aim to explore the association between cerebrospinal fluid tau protein levels (CSF-tau) and the severity of Alzheimer’s disease (AD). The E-value of 1.9262 preliminarily suggests the possible presence of hidden confounders in the data, even after adjusting for known confounders such as age, gender, education level, and deoxyribonucleic acid methylation (DNAm) levels. The sample precision matrix indicates correlations among variables to some extent. Therefore, previous methods may fail to address hidden confounding and inaccurately estimate causal relationships.

In this paper, we propose a deconfounded and debiased estimation for high-dimensional linear regression models with hidden confounding. Our estimator is developed via a two-step procedure involving spectral deconfounding and debiasing the bias from the weighted $\ell_{1}$ penalty. In the first phase, we apply the lasso method to the spectral transformed data to obtain a deconfounded estimator, where some leading singular values of the design matrix are shrunk. In the second phase, we debias the deconfounded estimator by inverting its Karush–Kuhn–Tucker conditions, where the inverse empirical covariance matrix of the spectral transformed design matrix is approximated through convex optimization programs. This novel debiasing approach does not require the sparse assumption on the precision matrix of the unconfounded component of the design matrix. It is more suitable for scenarios involving intrinsic correlations between covariates. In addition, the proposed method does not require any prior knowledge of hidden confounders.

In simulations, we consider the presence or absence of hidden confounders and the sparsity of the precision matrix. Simulation results demonstrate that the proposed estimator corrects the bias from hidden confounders and the weighted $\ell_{1}$ penalty and has a smaller variance. In the real data application, we focus on the association between CSF-tau and AD severity. The results indicate a positive association between CSF-tau levels and the severity of AD.

The rest of our paper is organized as follows. In Section Notation and model, we introduce notation and high-dimensional linear regression with hidden confounding. In Section Deconfounded and debiased estimation, we propose the deconfounded and debiased estimation and analyze the bias compared to other methods. We perform simulations and apply the proposed method to a DNAm dataset from the ADNI database in Sections Simulations and Real data application. Section Conclusion concludes the paper with discussions.

## 2 Notation and model

We start with an introduction to notation. For i=1,…,n and j=1,…,p, we use $Z_{i,j}$ to denote the (i,j) entry of the matrix $Z\in R^{n\times p}$, $Z_{\cdot j}\in R^{n}$ to denote the *j*th column vector, $Z_{\cdot ,-j}\in R^{n\times (p-1)}$ to denote the sub-matrix excluding the *j*th column vector, and $Z_{i\cdot }\in R^{p}$ to denote the transposition of the *i*th row vector. The $\ell_{\infty }$ norm of *Z* is denoted by $∥Z{∥}_{\infty }=\max_{i,j}|Z_{i,j}|$. For a set *Z*, |Z| denotes the cardinality of *Z*. Let [p]={1,…,p} and [n]={1,…,n}. We denote m=min{n,p}. The *I* denotes the identity matrix.

Given *n* i.i.d. samples consisting of covariates $X_{i\cdot }\in R^{p}$ and the response $Y_{i}\in R$ for i=1,…,n, we consider the high-dimensional linear regression with hidden confounding


(1) $$Y_{i}=\beta^{T}X_{i\cdot }+\phi^{T}H_{i\cdot }+e_{i},\;X_{i\cdot }=\Psi^{T}H_{i\cdot }+E_{i\cdot },$$

where $H_{i\cdot }\in R^{q}$ represents hidden confounders that are the common causes of $X_{i\cdot }$ and $Y_{i}$. The unconfounded component $E_{i\cdot }$ is independent of $H_{i\cdot }$. The random error $e_{i}$ is independent of $X_{i\cdot }$, $E_{i\cdot }$, and $H_{i\cdot }$. We assume that $e_{i}$ are sub-Gaussian with zero mean and variance $\sigma_{e}^{2}$. Denote the population covariance matrices of $E_{i\cdot }$ and $X_{i\cdot }$ as $\Sigma_{E}$ and $\Sigma_{X}$, respectively. Without loss of generality, it is assumed that $E(E_{i\cdot })=0$, $E(H_{i\cdot })=0$, $\text{Cov}(H_{i\cdot }^{T})=I_{q}$, and $\Sigma_{X}=\Psi^{T}\Psi +\Sigma_{E}$. The coefficients $\Psi \in R^{q\times p}$ and $\phi \in R^{q\times 1}$ encode the linear effect of hidden confounders $H_{i\cdot }$ on the measured covariates $X_{i\cdot }$ and the response $Y_{i}$. The coefficient of interest $\beta \in R^{p}$ quantifies the linear effect of the covariates on the response, which is assumed to be sparse.

To better illustrate the challenge of estimating the coefficient of interest β in the presence of hidden confounders, we rewrite (1) as the following perturbed linear regression


$$Y=X(\beta +b)+\epsilon ,\;\epsilon =e+H\phi -Xb,\;b=\Sigma_{X}^{-1}\Psi^{T}\phi ,$$

where $X={\left(X_{1\cdot },\ldots ,X_{n\cdot }\right)}^{T}\in R^{n\times p}$, $Y={\left(Y_{1},\ldots ,Y_{n}\right)}^{T}\in R^{n}$, $H={\left(H_{1\cdot },\ldots ,H_{n\cdot }\right)}^{T}\in R^{n\times q}$, and $\epsilon ={\left(\epsilon_{1},\ldots ,\epsilon_{n}\right)}^{T}\in R^{n}$. The variance of $\epsilon_{i}$ is $\sigma_{\epsilon }^{2}=\sigma_{e}^{2}+\phi^{T}(I-\Psi \Sigma_{X}^{-1}\Psi^{T})\phi$. The perturbation coefficient $b\in R^{p}$ captures the misleading effect of *X* on *Y* induced by hidden confounders. The definition of *b* leads to cov($X_{i\cdot },\phi^{T}H_{i\cdot }-b^{T}X_{i\cdot }$)=0, which implies that $X_{i\cdot }$ is uncorrelated with $\epsilon_{i}$. Estimating via some standard methods, like lasso, yields an estimator that captures the combined effect β+b, resulting in a significant bias. Distinguishing between β and *b* is challenging, making the accurate identification of β a non-trivial task.

## 3 Deconfounded and debiased estimation

To address hidden confounding in high-dimensional linear regression, we propose a two-step estimation: the first step corrects the hidden confounding bias through spectral transformation, and the second step reduces the bias incurred by the weighted $\ell_{1}$ penalty by the Karush–Kuhn–Tucker (KKT) conditions, ultimately deriving an analytical expression for the coefficient estimator. We give the outline of the proposed method in Algorithm 1, further analyze the bias of the proposed estimator, and compare it with other methods.

### 3.1 Step 1: deconfounding

The covariance decomposition $\Sigma_{X}=\Psi^{T}\Psi +\Sigma_{E}$ shows that $\Psi^{T}\Psi$ captures the hidden confounding, leading to inflation of the leading eigenvalues. Given that the singular values of the design matrix *X* scale with the square root of the eigenvalues of the covariance matrix $\Sigma_{X}$, hidden confounders inflate some top singular values of the design matrix (Ćevid *et al.* 2020). Thus, the perturbation term $∥Xb{∥}_{2}$ is large enough to be non-negligible, which will yield a biased estimator for β when directly regressing *Y* on *X*.

Spectral transformation is an effective method to reduce $∥Xb{∥}_{2}$ and thereby mitigate the bias caused by hidden confounders (Ćevid *et al.* 2020, Guo *et al.* 2022). The singular value decomposition of the design matrix yields $X=U\Lambda V^{T}$, where the left and right singular matrices $U\in R^{n\times m}$ and $V\in R^{p\times m}$ satisfy $U^{T}U=I$ and $V^{T}V=I$. The diagonal matrix $\Lambda \in R^{m\times m}$ contains the singular values of *X* in descending order, such that $\Lambda_{1,1}\geq \ldots \geq \Lambda_{m,m}$. The symmetric spectral transformation matrix is defined as


(2) $$Q=USU^{T},\;S_{i,i}=\{\begin{array}{cc} \Lambda_{\left\lfloor \rho m\right\rfloor ,\left\lfloor \rho m\right\rfloor }/\Lambda_{i,i} & \text{if}\;i\leq \left\lfloor \rho m\right\rfloor \\ 1 & \text{otherwise} \end{array}$$

where $S\in R^{m\times m}$ is a diagonal shrinkage matrix and $\Lambda_{\left\lfloor \rho m\right\rfloor ,\left\lfloor \rho m\right\rfloor }$ is the ρ-quantile of all singular values for a preassigned parameter ρ∈(0,1). There is a certain trade-off in choosing ρ: a smaller ρ, corresponding to more conservative shrinkage of *Q* which preserves more original information, yields a more efficient estimator. However, to effectively reduce the hidden confounding, the top few singular values of *X* must be sufficiently shrunk, necessitating ρm to sufficiently exceed the number of hidden confounders *q*. In practice, ρ=0.5 is typically used for balance (Guo *et al.* 2022). The spectral transformation matrix *Q* shrinks dominant singular values of *X* to the ρ-quantile $\Lambda_{\left\lfloor \rho m\right\rfloor ,\left\lfloor \rho m\right\rfloor }$. All singular values of *QX* exhibit significant attenuation compared to the original leading singular values of *X*. Specifically, the largest singular value of *QX* equals $\Lambda_{\left\lfloor \rho m\right\rfloor ,\left\lfloor \rho m\right\rfloor }$, and the remaining are less than $\Lambda_{\left\lfloor \rho m\right\rfloor ,\left\lfloor \rho m\right\rfloor }$. This transformation consequently reduces the magnitude of the perturbation term from $∥Xb{∥}_{2}$ to $∥\mathrm{QXb}{∥}_{2}$, rendering it negligible when regressing *QY* on *QX* via lasso.

We run the lasso on the spectral transformed data to reduce the hidden confounding. The deconfounded estimator is defined to be


(3) $${\hat{\beta }}^{\mathrm{dec}}=\arg\underset{\beta \in R^{p}}{\min}\{\frac{1}{2n}∥QY-QX\beta {∥}_{2}^{2}+\lambda \sum_{j=1}^{p}\frac{∥QX_{\cdot j}{∥}_{2}}{\sqrt{n}}|\beta_{j}|\},$$

where λ is a regularization parameter, typically determined through cross-validation. The deconfounded estimator is sparse due to the shrinkage effect of λ.

### 3.2 Step 2: debiasing

Despite mitigating the hidden confounding bias, the deconfounded estimator ${\hat{\beta }}^{\mathrm{dec}}$ inherits the bias incurred by the weighted $\ell_{1}$ penalty. To proceed to debias ${\hat{\beta }}^{\mathrm{dec}}$, Guo *et al.* (2022) adopted the debiased lasso method proposed by Zhang and Zhang (2014) under a sparse assumption of the precision matrix of $E_{i\cdot }$. We aim to extend the method, which can correct both the hidden confounding bias and the bias incurred by the weighted $\ell_{1}$ penalty, to accommodate a broader range of precision matrices, such as non-sparse cases. Inspired by Javanmard and Montanari (2014), we invert the Karush–Kuhn–Tucker (KKT) conditions of the deconfounded estimator and propose a deconfounded and debiased estimation.

By the KKT conditions, ${\hat{\beta }}^{\mathrm{dec}}$ satisfies


$$-\frac{{\left(QX\right)}^{T}(QY-QX{\hat{\beta }}^{\mathrm{dec}})}{n}+\lambda \hat{K}=0,$$

where $\hat{K}={\left({\hat{K}}_{1},\ldots ,{\hat{K}}_{p}\right)}^{T}$ with ${\hat{K}}_{j}$ denoting the subdifferential of $∥QX_{\cdot j}{∥}_{2}|\beta_{j}|/\sqrt{n}$ with respect to $\beta_{j}$ for j=1,…,p. Denote the empirical covariance matrix of *QX* by $\hat{\Sigma }={\left(QX\right)}^{T}(QX)/n$. After inverting the KKT conditions, we derive


(4) $$\hat{\Sigma }({\hat{\beta }}^{\mathrm{dec}}-\beta )+\lambda \hat{K}=\frac{{\left(QX\right)}^{T}Q\epsilon }{n}+\hat{\Sigma }b.$$

Suppose that $\hat{\Theta }\in R^{p\times p}$ is an approximate inverse of $\hat{\Sigma }$. Multiplying both sides of (4) by $\hat{\Theta }$, we arrive at the following expression


(5) $$\begin{array}{c} {\hat{\beta }}^{\mathrm{dec}}-\beta +\frac{\hat{\Theta }{\left(QX\right)}^{T}(QY-QX{\hat{\beta }}^{\mathrm{dec}})}{n}\\ =\frac{\hat{\Theta }{\left(QX\right)}^{T}Q\epsilon }{n}+\hat{\Theta }\hat{\Sigma }b+(I-\hat{\Theta }\hat{\Sigma })({\hat{\beta }}^{\mathrm{dec}}-\beta ). \end{array}$$

Consequently, by (5), we propose the deconfounded and debiased estimator as follows:


(6) $$\hat{\beta }={\hat{\beta }}^{\mathrm{dec}}+\frac{\hat{\Theta }{\left(QX\right)}^{T}(QY-QX{\hat{\beta }}^{\mathrm{dec}})}{n}.$$

The debiasing performance of $\hat{\beta }$ critically depends on the approximate inverse empirical covariance matrix $\hat{\Theta }$. A well-designed $\hat{\Theta }$ should simultaneously minimize the variance of $\hat{\beta }$ and control its bias. Thus, we propose constructing $\hat{\Theta }$ by minimizing the stochastic variance $\text{Var}(\hat{\Theta }{\left(QX\right)}^{T}Q\epsilon /n)=\sigma_{\epsilon }^{2}\hat{\Theta }X^{T}Q^{4}X{\hat{\Theta }}^{T}/n^{2}$, subject to bias control constraint $∥I-\hat{\Theta }\hat{\Sigma }{∥}_{\infty }\leq \mu$, where μ>0 is a preassigned tolerance parameter for the maximum entry-wise deviation. The matrix-wise convex optimization program for $\hat{\Theta }$ is


(7) $$\hat{\Theta }=\arg\underset{\Theta \in R^{p\times p}}{\min}\{\Theta X^{T}Q^{4}X\Theta^{T}:∥I-\Theta \hat{\Sigma }{∥}_{\infty }\leq \mu \}.$$

Given $\Theta ={\left(\theta_{1},\ldots ,\theta_{p}\right)}^{T}$, (7) is rewritten as *p* vector-wise convex optimization programs for j=1,…,p:


(8) $$\begin{array}{cc} \hat{\theta_{j}} & =\arg\underset{\theta_{j}\in R^{p}}{\min}\{\theta_{j}^{T}X^{T}Q^{4}X\theta_{j}:∥I_{\cdot j}-\hat{\Sigma }\theta_{j}{∥}_{\infty }\leq \mu \},\\ \hat{\Theta } & ={\left({\hat{\theta }}_{1},\ldots ,{\hat{\theta }}_{p}\right)}^{T}. \end{array}$$

If any of *p* convex optimization programs are infeasible, we set $\hat{\Theta }=I$. To solve these optimization programs, we apply the equivalent Lagrange dual problem, a technique that is frequently used (Javanmard and Montanari, 2014, Guo *et al.* 2019). Despite the above focus on high-dimensional linear regression with hidden confounding, the proposed method is versatile enough to address the low-dimensional setting.

### 3.3 Bias analysis

Algorithm 1 outlines the complete procedure for the deconfounded and debiased estimation $\hat{\beta }$. The error decomposition of $\hat{\beta }$ is


(9) $$\hat{\beta }-\beta =\underset{\text{variability}}{\underset{︸}{\frac{1}{n}\hat{\Theta }{\left(QX\right)}^{T}Q\epsilon }}+\underset{\;\text{remaining}\;\text{bias}}{\underset{︸}{\hat{\Theta }\hat{\Sigma }b+(I-\hat{\Theta }\hat{\Sigma })({\hat{\beta }}^{\mathrm{dec}}-\beta )}}.$$

The first term on the right-hand side of (9) represents the stochastic variability of $\hat{\beta }$. The bias after deconfounding and debiasing encapsulates two components: the remaining hidden confounding bias and the remaining bias due to the estimation error of ${\hat{\beta }}^{\mathrm{dec}}$.

Algorithm 1 Outline of the deconfounded and debiased estimation

**Require**: Design matrix *X*, response *Y*, and parameters ρ, λ, and μ

**Ensure:** Deconfounded and debiased estimator $\hat{\beta }$

1: Construct the spectral transformation matrix *Q* as (2)

2: Compute the deconfounded estimator ${\hat{\beta }}^{\mathrm{dec}}$ as (3)

3: Solve the matrix $\hat{\Theta }$ by convex optimization programs (8)

4: Compute the deconfounded and debiased estimator $\hat{\beta }$ as (6)

Focusing on an individual component of the proposed estimator, ${\hat{\beta }}_{j}$ for j∈[p], we impose upper bounds on the remaining bias


$$\begin{array}{cc} & |{\left(\hat{\Theta }\hat{\Sigma }\right)}_{j,\cdot }b|\leq ∥{\left(\hat{\Theta }\hat{\Sigma }\right)}_{j,-j}{∥}_{\infty }∥b_{-j}{∥}_{1}+|{\left(\hat{\Theta }\hat{\Sigma }\right)}_{j,j}||b_{j}|,\\ & |{\left(I-\hat{\Theta }\hat{\Sigma }\right)}_{j,\cdot }({\hat{\beta }}^{\mathrm{dec}}-\beta )|\leq ∥{\left(I-\hat{\Theta }\hat{\Sigma }\right)}_{j,\cdot }{∥}_{\infty }∥{\hat{\beta }}^{\mathrm{dec}}-\beta {∥}_{1}. \end{array}$$

By the matrix-wise convex optimization program (7), we have $∥{\left(I-\hat{\Theta }\hat{\Sigma }\right)}_{j,\cdot }{∥}_{\infty }\leq \;\mu \;$, $∥{\left(\hat{\Theta }\hat{\Sigma }\right)}_{j,-j}{∥}_{\infty }\leq \;\mu \;$, and $|{\left(\hat{\Theta }\hat{\Sigma }\right)}_{j,j}|\leq \;\mu \;+1$. The parameter μ limits the difference between $\hat{\Theta }\hat{\Sigma }$ and the identity matrix, so μ is a given small value which dramatically shrinks the upper bounds of the remaining bias. In the deconfounding step, we shrink *Xb* through the spectral transformation matrix, ensuring that $∥\mathrm{QXb}{∥}_{2}$ is small enough to be negligible. Thus, compared with the lasso method (Tibshirani, 1996) and the debiased lasso methods that cannot address the hidden confounding (Javanmard and Montanari, 2014, Van de Geer *et al.* 2014, Zhang and Zhang, 2014), the deconfounded estimator ${\hat{\beta }}^{\mathrm{dec}}$ achieves smaller estimation error.

### 3.4 Method comparisons

We sequentially compare our method with Guo *et al.* (2022) and Sun *et al.* (2024), both of which are capable of achieving deconfounding and debiasing, to illustrate several advantages of our method.

- The main difference between our method and Guo *et al.* (2022) is the debiasing strategy. Whereas Guo *et al.* (2022) adopts low-dimensional projections based on node-wise regressions akin to Zhang and Zhang (2014), we invert the Karush–Kuhn–Tucker conditions and employ optimization-based precision matrix estimation, inspired by Javanmard and Montanari (2014). This eliminates the need for the sparse precision matrix assumption in Guo *et al.* (2022), thereby relaxing the restrictions on intrinsic correlations between covariates.
- The method in Sun *et al.* (2024) diverges from ours in both debiasing and deconfounding. It shares the debiasing procedure in Guo *et al.* (2022) and Zhang and Zhang (2014), reaffirming our advantage in avoiding the sparse assumption. For deconfounding, Sun *et al.* (2024) introduces a diagonal shrinkage matrix *S* with $S_{i,i}=I(i>q)$, necessitating consistent estimation of the unknown number of hidden confounders *q*. Inconsistent estimation may degrade estimator performance. In contrast, our diagonal shrinkage matrix *S*, following the similar idea of Ćevid *et al.* (2020) and Guo *et al.* (2022), only requires a preassigned quantile parameter ρ that ensures the aforementioned trade-off balance (typically ρ=0.5). We completely avoid auxiliary dimension estimation.

It seems that our method appears computationally intensive due to the optimization program (7) in high-dimensional settings. Actually, solving (7) is not more burdensome than solving node-wise regressions in Guo *et al.* (2022) and Sun *et al.* (2024). This can be confirmed by checking that the dual of (7) is an $\ell_{1}$-regularized quadratic optimization problem.

Many methods for deconfounding rely on information about hidden confounders (Wang *et al.* 2017, Gold *et al.* 2020, Yang and Ding 2020). For example, the two-stage calibration method utilizes an additional dataset, containing information on hidden confounders missing in the main dataset, to correct the confounding in the main dataset (Yang and Ding 2020). In contrast, the proposed method does not require knowledge of hidden confounders. It directly shrinks the confounding by the spectral transformation matrix *Q*, effectively reducing the hidden confounding bias in the coefficient estimates.

## 4 Simulations

In this section, we conduct simulation studies to compare the finite-sample performance of the proposed method with some existing approaches. Our method is compared against (i) the debiased lasso (DL) method (Javanmard and Montanari 2014), which cannot handle the hidden confounding, and (ii) the doubly debiased lasso (DDL) method (Guo *et al.* 2022), which uses a different debiasing strategy than we do. The proposed estimation involves three parameters to be determined. The regularization parameter λ for the deconfounded estimator is determined via the 10-fold cross-validation. Guided by Javanmard and Montanari (2014), the parameter μ in convex optimization programs is set as $0.5\sqrt{(\text{log}\;p)/n}$. Regarding the quantile ρ in the spectral transformation matrix *Q*, we perform simulations to investigate its impact on method performance (detailed in Section 2, available as supplementary data at *Bioinformatics* online). Our empirical results support setting ρ=0.5, consistent with the parameter setting in Guo *et al.* (2022).

Entries of hidden confounders *H*, the random error *e*, and the coefficient ϕ are independently generated from N(0,1), and the coefficient Ψ is set to have independent N(1,1) entries. Rows of the unconfounded component *E* are independently generated from the multivariate normal distribution with mean zero and covariance matrix $\Sigma_{E}(\varrho )$, where $\Sigma_{E}(\varrho )$ has a compound symmetric correlation structure with the correlation parameter ϱ. The correlation parameter ϱ influences the strength of correlation among covariates. The coefficient of interest is fixed as $\beta ={\left(\beta_{1},\beta_{2},\ldots ,\beta_{p}\right)}^{T}={\left(1,1,1,1,1,0,\ldots ,0\right)}^{T}$, and the number of hidden confounders is set as q=3.

We consider three different scenarios:

Scenario 1 (non-sparse precision matrix and the presence of hidden confounders). The parameter of the covariance matrix $\Sigma_{E}(\varrho )$ is set as ϱ=0.5, and its corresponding precision matrix is non-sparse. In the presence of hidden confounders, the design matrix is constructed as X=HΨ+E and the response is constructed as Y=Xβ+Hϕ+e.

Scenario 2 (sparse precision matrix and the presence of hidden confounders). The parameter of the covariance matrix $\Sigma_{E}(\varrho )$ is set as ϱ=0, and its corresponding precision matrix is sparse. In the presence of hidden confounders, the design matrix and response are constructed as in scenario 1.

Scenario 3 (non-sparse precision matrix and the absence of hidden confounders). The parameter of the covariance matrix $\Sigma_{E}(\varrho )$ is set as ϱ=0.5, and its corresponding precision matrix is non-sparse. In the absence of hidden confounders, the design matrix is constructed as X=E and the response is constructed as Y=Xβ+Hϕ+e.

To better demonstrate the ability of the proposed method in bias correction, we conduct all simulations under high-dimensional settings n<p, as the bias introduced by the weighted $\ell_{1}$ penalty becomes more pronounced in such settings. For the estimation and inference of the individual coefficient $\beta_{1}=1$, we calculate bias (BIAS), root mean square error (RMSE), and standard error (SE). We consider different configurations of (n,p,ϱ), and each configuration is independently repeated 1000 times.

Tables 1 and 2 show the simulation results for scenario 1 and scenario 2 across different *n* and *p*, respectively. Both the proposed and DDL methods demonstrate negligible bias across varying sample sizes *n* and dimensions *p* for both sparse and non-sparse precision matrices. This indicates their effectiveness in correcting the bias from hidden confounders and the weighted $\ell_{1}$ penalty. In contrast, the DL method exhibits significant bias, primarily attributable to hidden confounders. Besides, the proposed method shows the smallest SE, suggesting a more precise coefficient estimation. The DDL method exhibits a smaller bias compared to the proposed method, but its SE is larger. This reflects its characteristic of sacrificing variance for better bias, as shown in Guo *et al.* (2022). The proposed method achieves smaller variance while maintaining bias control, resulting in the smallest RMSE. As the sample size *n* increases, the RMSE, BIAS, and SE of the proposed method decrease, and the bias gap between the proposed and DDL methods gradually narrows. In the absence of hidden confounders, we evaluate the performance of the three methods under (n,p,ϱ)=(500,600,0.5). As depicted in Table 3, the proposed estimator also demonstrates proficiency in rectifying bias from the weighted $\ell_{1}$ penalty.

**Table 1.** Performance of BIAS, RMSE, and SE under varying *n* and *p*, where ϱ=0.5 indicates the non-sparse case of the precision matrix of $E_{i\cdot }$.

(n,p,ϱ)	Method	BIAS	RMSE	SE
(500,600,0.5)	Proposed	−0.0154	0.0717	0.0701
	DDL	0.0021	0.0910	0.0911
	DL	−0.1755	0.2172	0.1280
(500,800,0.5)	Proposed	−0.0147	0.0728	0.0714
	DDL	0.0041	0.0896	0.0895
	DL	−0.1832	0.2256	0.1317
(500,1000,0.5)	Proposed	−0.0211	0.0766	0.0737
	DDL	0.0025	0.0915	0.0915
	DL	−0.1862	0.2241	0.1247
(700,1000,0.5)	Proposed	−0.0114	0.0594	0.0583
	DDL	−0.0013	0.0744	0.0745
	DL	−0.1790	0.2200	0.1281
(900,1000,0.5)	Proposed	−0.0091	0.0505	0.0497
	DDL	−0.0020	0.0627	0.0627
	DL	−0.1734	0.2137	0.1250

DDL: doubly debiased lasso method (Guo *et al.* 2022); DL: debiased lasso method (Javanmard and Montanari, 2014).

**Table 2.** Performance of BIAS, RMSE, and SE under varying *n* and *p*, where ϱ=0 indicates the sparse case of the precision matrix of $E_{i\cdot }$.

(n,p,ϱ)	Method	BIAS	RMSE	SE
(500,600,0)	Proposed	−0.0086	0.0504	0.0497
	DDL	0.0010	0.0634	0.0634
	DL	−0.1023	0.1366	0.0907
(500,1000,0)	Proposed	−0.0097	0.0536	0.0528
	DDL	0.0023	0.0643	0.0643
	DL	−0.1084	0.1395	0.0879
(900,1000,0)	Proposed	−0.0057	0.0340	0.0336
	DDL	−0.0003	0.0437	0.0437
	DL	−0.1018	0.1317	0.0837

DDL: doubly debiased lasso method (Guo *et al.* 2022); DL: debiased lasso method (Javanmard and Montanari, 2014).

**Table 3.** Performance of BIAS, RMSE, and SE in the absence of hidden confounders fixing (n,p,ϱ)=(500,600,0.5).

Method	BIAS	RMSE	SE
Proposed	−0.0140	0.1368	0.1361
DDL	−0.0052	0.1834	0.1835
DL	−0.1042	0.1691	0.1332

DDL: doubly debiased lasso method (Guo *et al.* 2022); DL: debiased lasso method (Javanmard and Montanari, 2014).

## 5 Real data application

We apply the proposed method to a real-world dataset from the Alzheimer’s Disease Neuroimaging Initiative (ADNI) database, a publicly available resource focused on Alzheimer’s disease (AD) research. Some medical studies have demonstrated an association between cerebrospinal fluid tau protein levels (CSF-tau) and the severity of AD (Braak *et al.* 2011, Jack Jr et al. 2018, Shaw *et al.* 2018, Mummery *et al.* 2023). Under normal conditions, tau protein plays a critical role in stabilizing microtubule structures (Kametani and Hasegawa 2018). When tau protein undergoes abnormal phosphorylation and aggregates into neurofibrillary tangles, it disrupts neuronal stability and function, triggering the pathological cascade of AD and ultimately leading to dementia (Iqbal *et al.* 2010). Following neuronal damage or death, tau protein is released into the extracellular space and enters the cerebrospinal fluid (CSF). CSF-tau can activate microglia and release inflammatory mediators, further damaging surrounding neurons and synapses and promoting cognitive impairment (Mummery *et al.* 2023). Therefore, we aim to explore the potential influence of CSF-tau on AD severity, supporting the development of drugs targeting CSF-tau, personalized AD treatment strategies, and early identification and intervention for AD.

The real-world dataset includes 313 participants with complete records of exposure, outcome, and candidate covariates. The exposure of interest, CSF-tau, is measured at baseline. The outcome, the severity of AD, is evaluated by the widely accepted 11-item version of Alzheimer’s disease Assessment Scale (ADAS-11) cognitive score, measured at month 24. The ADAS-11 score ranges from 0 to 70, with a higher score indicating greater severity. Age, gender, and education level are well-documented risk factors for AD (Vina and Lloret 2010). Therefore, these three factors are considered as candidate covariates in our study. Zhang *et al.* (2023) identify distinct deoxyribonucleic acid methylation (DNAm) patterns in CSF biomarkers between individuals with AD and cognitively normal controls. Thus, we pre-process the DNAm levels of whole-genome CpG sites by five steps: (i) excluding probes with *P*-value <0.05, (ii) filtering out gender-related probes, (iii) removing probes with SNPs at CpG sites, (iv) eliminating cross-reactive probes, and (v) averaging DNAm levels for samples measured multiple times (Shireby *et al.* 2020). After these pre-processing steps, we retained the DNAm levels at 865860 CpG sites as the rest candidate covariates in the real-world dataset.

**Figure 1.**
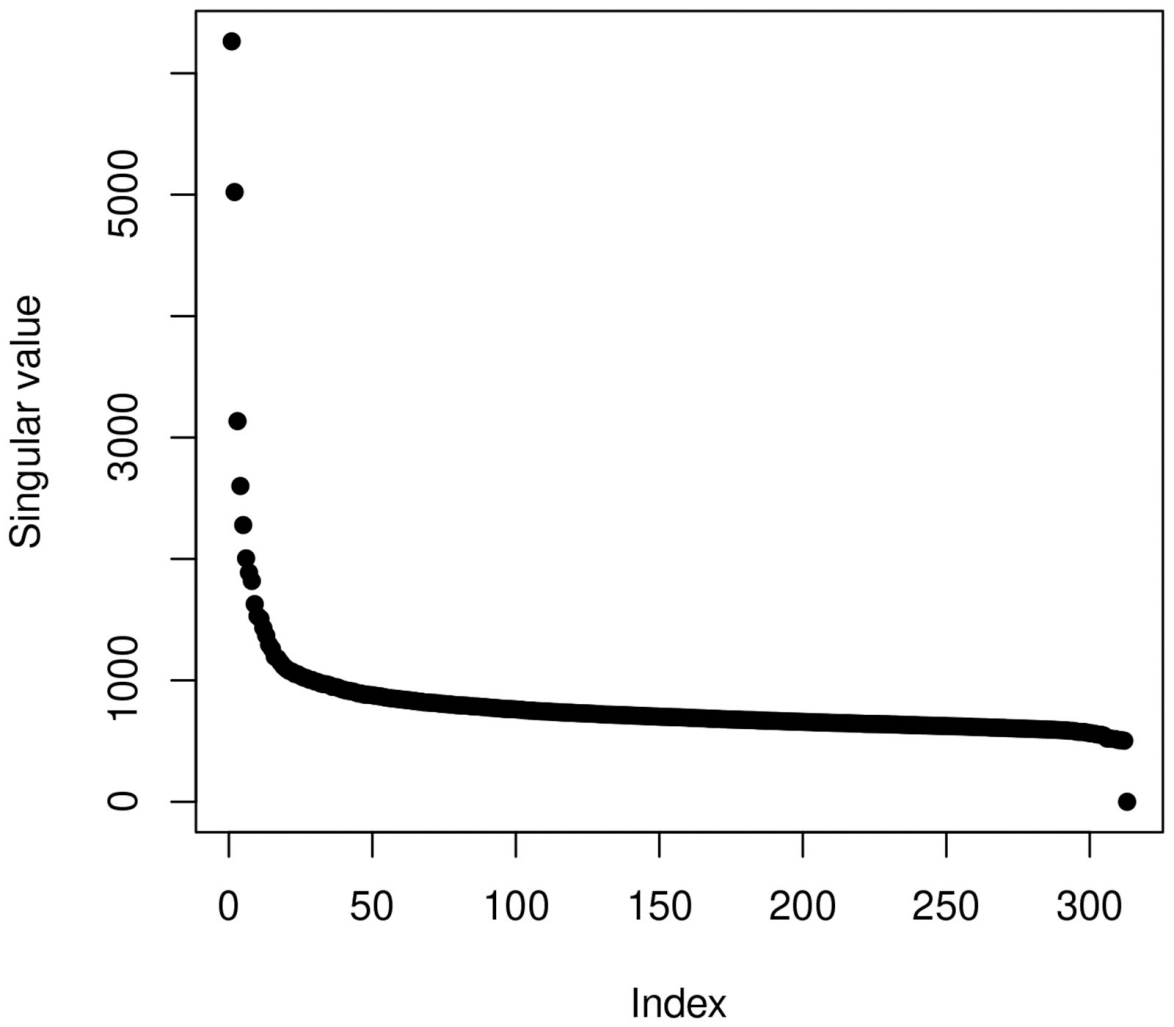
Singular values of the exposure-covariate matrix on the standardized dataset.

We first standardized the dataset to eliminate the influence of differences in scale and magnitude in the data. To detect hidden confounders, we calculate and plot the singular values of the exposure-covariate matrix, as their presence manifests as inflated leading singular values (Ćevid *et al.* 2020). Figure 1 shows that top singular values are substantially larger than the rest, indicating that the exposure and covariates are influenced by hidden confounders (Guo *et al.* 2022).

**Figure 2.**
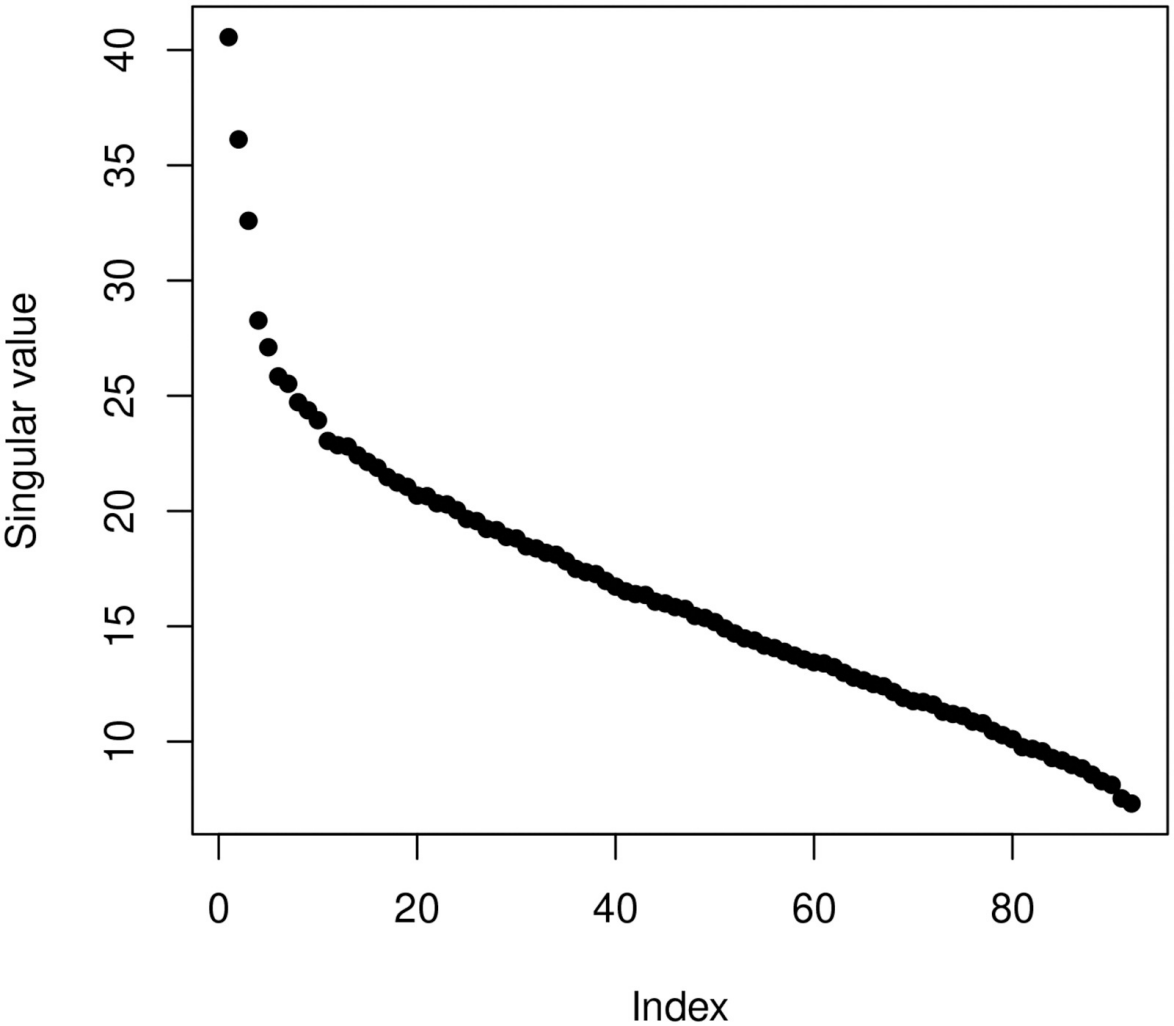
Singular values of the exposure-covariate matrix on the standardized pre-selected dataset.

**Figure 3.**
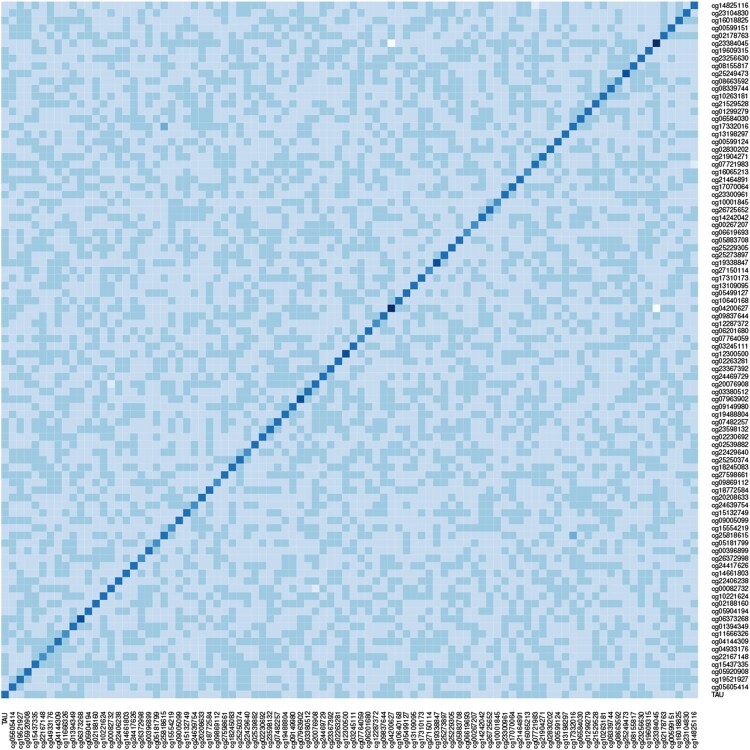
The sample precision matrix of the standardized pre-selected dataset.

Due to multicollinearity among covariates, we pre-select 91 CpG sites by the lasso method. The DNAm levels at 91 CpG sites, along with the CSF-tau and the ADAS-11 score form the pre-selected dataset. After the standardization, we calculate the singular values on the standardized pre-selected dataset, as shown in Figure 2. Furthermore, we also provide the E-value of 1.9262 to indicate that hidden confounding still exists in this dataset. Additionally, as shown in Figure 3, we calculate the sample precision matrix, suggesting that the population precision matrix among variables is likely non-sparse. These findings support the use of the proposed method to explore the association between CSF-tau and AD severity, potentially yielding more accurate results.

On the standardized pre-selected dataset, we estimate the coefficient of CSF-tau and compute its 95% confidence interval based on 500 bootstrap replications. The methods compared with the proposed one include the doubly debiased lasso (DDL) method (Guo *et al.* 2022), the debiased lasso (DL) method (Javanmard and Montanari 2014), and the lasso method (Tibshirani 1996). Parameters in all methods are consistent with the settings used in simulations. As shown in Table 4, CSF-tau levels are positively associated with AD severity, which remains significant after deconfounding. This finding supports the critical role of CSF-tau in AD pathology and may provide insights for early diagnosis and disease monitoring. The proposed method indicates that holding other variables constant, a one-unit increase in CSF-tau is associated with an average increase of 0.2938 units in the ADAS-11 score. The DDL method corrects both the hidden confounding bias and the bias incurred by the $\ell_{1}$ penalty under a sparse precision matrix assumption. Figure 3 suggests that the sparsity assumption may not hold for our dataset. This could explain why the coefficient estimate (0.2890) from the DDL method in Table 4 is smaller than that (0.2938) from the proposed method and closer to the estimates (0.2889; 0.2892) from the DL and lasso methods. The DL method cannot handle the hidden confounding bias, while the lasso method fails to correct both biases. Additionally, compared to the DDL and DL methods, our proposed method yields a narrower confidence interval (0.2494, 0.3488).

**Table 4.** Coefficient estimates of CSF-tau and 95% confidence intervals based on 500 bootstrap replications.

Methods	Estimates	95% confidence intervals
Proposed	0.2938	(0.2494, 0.3488)
DDL	0.2890	(0.2422, 0.3461)
DL	0.2889	(0.2429, 0.3468)
Lasso	0.2892	(0.2436, 0.3407)

DDL: doubly debiased lasso method (Guo *et al.* 2022); DL: debiased lasso method (Javanmard and Montanari, 2014); Lasso, lasso method (Tibshirani, 1996).

## 6 Conclusion

In this article, we propose a deconfounded and debiased estimation by two steps: spectral deconfounding and correcting the bias from the weighted $\ell_{1}$ penalty. We first obtain a deconfounded estimator by spectral transformation, then debias the deconfounded estimator by inverting its Karush–Kuhn–Tucker conditions and solving convex optimization programs. The advantage of the proposed method lies in its independence from the sparse assumption on the precision matrix of the unconfounded component of the design matrix, allowing for broader applicability to data with intrinsic correlations between covariates. Unlike previous methods, the proposed method does not require any prior information on hidden confounders. In simulations, the proposed estimator corrects the bias from hidden confounders and the weighted $\ell_{1}$ penalty and has a more precise coefficient estimation, under the presence or absence of hidden confounders and different sparsity of the precision matrix. In the analysis of the DNAm dataset from the ADNI database, we obtain a positive association between CSF-tau levels and AD severity.

We only consider the linear regression with continuous outcomes in this paper. This approach may be extended to generalized linear models for categorical outcomes, Cox models for survival data, and quantile regression models for skewed distributed outcomes. The challenge, however, is that the practical feasibility and theoretical justification of the spectral transformation for deconfounding in nonlinear models remain unclear. These questions are highly worthy of research and will be the focus of our future research.

## Supplementary Material

btaf400_Supplementary_Data

## Data Availability

The data underlying this article are available in the Alzheimer's Disease Neuroimaging Initiative (ADNI) database at https://adni.loni.usc.edu/.
