# Supplementary material for: Deconfounded and debiased estimation for high-dimensional linear regression under hidden confounding with application to omics data
Source: Bioinformatics. 2025 Jul 14;41(7):btaf400. doi: 10.1093/bioinformatics/btaf400 (PMC12381636; doi:10.1093/bioinformatics/btaf400)
Supplement: btaf400_Supplementary_Data [file btaf400_supplementary_data.pdf]

Supplementary material for *"Deconfounded and debiased estimation for high-dimensional linear regression under hidden confounding with application to omics data"*

S1 Derivation Details

We provide the detailed derivation process of equation (4) to equation (5). After inverting the KKT conditions, we derive the equation (4)

$$\begin{aligned}\hat{\Sigma}(\hat{\beta}^{dec}-\beta)+\lambda\hat{K} &= \frac{(QX)^{\mathrm{T}}(QX)(\hat{\beta}^{dec}-\beta)}{n}+\frac{(QX)^{\mathrm{T}}(QY-QX\hat{\beta}^{dec})}{n}\\ &= \frac{(QX)^{\mathrm{T}}Q\epsilon}{n}+\hat{\Sigma}b.\end{aligned}$$

Suppose that $\hat{\Theta}\in\mathbb{R}^{p\times p}$ is an approximate inverse of $\hat{\Sigma}$. Multiplying both sides of the above equation by $\hat{\Theta}$, we have

$$\hat{\Theta}\hat{\Sigma}(\hat{\beta}^{dec}-\beta)+\frac{\hat{\Theta}(QX)^{\mathrm{T}}(QY-QX\hat{\beta}^{dec})}{n}=\frac{\hat{\Theta}(QX)^{\mathrm{T}}Q\epsilon}{n}+\hat{\Theta}\hat{\Sigma}b.$$

Through straightforward simplification, we arrive at the equation (5)

$$\begin{aligned}\hat{\beta}^{dec}-\beta+\frac{\hat{\Theta}(QX)^{\mathrm{T}}(QY-QX\hat{\beta}^{dec})}{n} &= (\hat{\beta}^{dec}-\beta)-\hat{\Theta}\hat{\Sigma}(\hat{\beta}^{dec}-\beta)+\frac{\hat{\Theta}(QX)^{\mathrm{T}}Q\epsilon}{n}+\hat{\Theta}\hat{\Sigma}b\\ &= \frac{\hat{\Theta}(QX)^{\mathrm{T}}Q\epsilon}{n}+\hat{\Theta}\hat{\Sigma}b+(I-\hat{\Theta}\hat{\Sigma})(\hat{\beta}^{dec}-\beta).\end{aligned}$$

S2 Additional simulations

To investigate the effect of ρ on the performance of the proposed method, we conduct simulations under scenario 1 and scenario 2. We fix $n=250$ and $p=300$ while varying $\rho\in\{0.1,0.3,0.5,0.7,0.9\}$, with 100 independent replications per setting. As shown in Table 1, our method achieves the smallest BIAS when $\rho=0.5$. Aligning with the aforementioned trade-off analysis of ρ, a larger ρ leads to a larger SE. Since the BIAS is relatively smaller in magnitude compared to the SE, the RMSE increases monotonically with ρ. Thus, following Guo et al. (2022), we also recommend $\rho=0.5$ as the default value for all subsequent simulations.

Table 1: Our method's performance of BIAS, RMSE, and SE under scenario 1 and scenario 2 varying $\rho \in \{0.1, 0.3, 0.5, 0.7, 0.9\}$.

(n, p, ϱ)	ρ	BIAS	RMSE	SE
(250,300,0.5)	0.1	-0.0105	0.1044	0.1044
	0.3	-0.0095	0.1052	0.1053
	0.5	-0.0091	0.1067	0.1069
	0.7	-0.0118	0.1104	0.1103
	0.9	-0.0164	0.1176	0.1170
(250,300,0)	0.1	-0.0223	0.0738	0.0707
	0.3	-0.0212	0.0741	0.0714
	0.5	-0.0205	0.0748	0.0723
	0.7	-0.0222	0.0765	0.0736
	0.9	-0.0284	0.0814	0.0767

References

Guo, Z., Ćevid, D., and Bühlmann, P. (2022). Doubly debiased lasso: High-dimensional inference under hidden confounding. Annals of statistics, 50(3):1320.
